# Evaluating the Performance of Pre-Trained Convolutional Neural Network for Audio Classification on Embedded Systems for Anomaly Detection in Smart Cities

**DOI:** 10.3390/s23136227

**Published:** 2023-07-07

**Authors:** Mimoun Lamrini, Mohamed Yassin Chkouri, Abdellah Touhafi

**Affiliations:** 1 Department of Engineering Sciences and Technology (INDI), Vrije Universiteit Brussel (VUB), 1050 Brussels, Belgium; abdellah.touhafi@vub.be; 2 SIGL Laboratory, National School of Applied Sciences of Tetuan, Abdelmalek Essaadi University, Tetuan 93000, Morocco; mychkouri@uae.ac.ma; 3 Department of Electronics and Informatics (ETRO), Vrije Universiteit Brussel (VUB), 1050 Brussels, Belgium

**Keywords:** deep learning, pre-trained models, environment sound recognition, embedded system

## Abstract

Environmental Sound Recognition (ESR) plays a crucial role in smart cities by accurately categorizing audio using well-trained Machine Learning (ML) classifiers. This application is particularly valuable for cities that analyzed environmental sounds to gain insight and data. However, deploying deep learning (DL) models on resource-constrained embedded devices, such as Raspberry Pi (RPi) or Tensor Processing Units (TPUs), poses challenges. In this work, an evaluation of an existing pre-trained model for deployment on Raspberry Pi (RPi) and TPU platforms other than a laptop is proposed. We explored the impact of the retraining parameters and compared the sound classification performance across three datasets: ESC-10, BDLib, and Urban Sound. Our results demonstrate the effectiveness of the pre-trained model for transfer learning in embedded systems. On laptops, the accuracy rates reached 96.6% for ESC-10, 100% for BDLib, and 99% for Urban Sound. On RPi, the accuracy rates were 96.4% for ESC-10, 100% for BDLib, and 95.3% for Urban Sound, while on RPi with Coral TPU, the rates were 95.7% for ESC-10, 100% for BDLib and 95.4% for the Urban Sound. Utilizing pre-trained models reduces the computational requirements, enabling faster inference. Leveraging pre-trained models in embedded systems accelerates the development, deployment, and performance of various real-time applications.

## 1. Introduction

ESR is a critical component for various applications, such as noise pollution monitoring, public safety [1], industrial safety, and smart cities [2]. Environmental noise monitoring is conducted to detect and track the sources of disturbance in residential and natural environments, including the sound produced by aircraft during takeoff and landing at nearby airports [3]. Intelligent sound monitoring devices are commonly deployed using embedded platforms, such as microcontrollers or digital signal processors. Various methods, including Machine Learning algorithms and signal processing techniques, have been investigated to achieve the accurate classification of urban sounds with a limited number of embedded devices. Several studies have employed the classical ML algorithms, such as k-Nearest Neighbor (k-NN), Support Vector Machine (SVM), Naive Bayes, and Decision Trees, which have been previously evaluated [4]. Sound recognition has seen significant progress in ML, as evidenced by the recent advancements. Specifically, ANN and CNN have been shown to offer precise sound recognition with high accuracy levels [5]. Nonetheless, when these methods are applied to embedded devices, there is a reduction in the accuracy and longer inference times. This is due to the need for computationally intensive operations, which are often limited by the available resources of embedded systems. Capturing audio using a microphone on-site and analyzing it in the cloud may not always be feasible or preferable. This method not only raises privacy concerns due to potential interception by a third party but also results in significant latency.

There are existing specialized platforms available for this purpose [6]. For instance, Google has developed an extension to its well-known machine learning framework TensorFlow (TF), which allows for rapid and efficient inference of DL on advanced embedded platforms such as RPi. In addition, Google has developed a specialized hardware accelerator exclusively designed for deep neural networks, known as the TPU, which serves as an embedded solution with low latency and low power consumption.

The work presented in this paper assesses the current methods for implementing a pre-trained model on embedded systems. The pre-trained model for ESR has been embedded as an example. Due to the demands of low latency, power efficiency, and real-time response required by the application, the evaluation focuses on low-end TPUs as embedded devices, whereas high-performance general-purpose embedded devices such as the RPi are employed as a benchmark or comparison point. The evaluation of hardware accelerators, specifically TPUs, was conducted from both accuracy and latency perspectives. Additionally, various tool flows are examined, evaluated, and discussed in terms of embedding pre-trained models, including Edge TPU [7] and TensorFlow Lite (TFLite) [8]. This comprehensive analysis offers valuable insights into the necessary steps and effort required for embedding pre-trained models on TPUs as well as the different strategies employed to use these technologies.

The principal achievements of this study can be summarized as follows:

- A technique to evaluate audio features based on windowing, aimed to increase the size and diversity of a training datasets;
- Combination of CNN and ANN based on DL with a pre-trained model and evaluation of their deployment on embedded devices for ESR;
- An in-depth examination, assessment, and discussion of existing tool flows for deploying a pre-trained model on TPUs;
- A comparison of the performance attainable on TPUs for ESR using a combination of a pre-trained model with CNN and ANN models.

Our proposed system represents a significant advancement in utilizing a pre-trained model to replicate the performance achieved on laptops. In addition to introducing our innovative methodology, we present two DL models specifically designed to address the constraints imposed by embedded devices. To thoroughly assess and compare the effectiveness of both approaches, we conduct experiments across different platforms, including a standard PC and a widely used embedded device. To ensure a fair and comprehensive evaluation, we leverage multiple audio datasets commonly used in related studies. Our evaluation process focuses on key metrics such as accuracy and execution time, which provide valuable insights into the performance of our models.

Time-series classification refers to the task of categorizing or labeling time-series data into different classes or categories based on their temporal patterns. In this problem, the input data consists of sequences of observations or measurements taken at regular intervals over time. Two important aspects of the time-series classification problem are feature extraction and evaluation metrics. Feature extraction plays a critical role in time-series classification. The objective is to identify meaningful features or representations that capture the distinctive patterns in the data. Techniques such as Fourier transforms, wavelet transforms, or feature engineering can be employed to achieve this goal. When assessing the performance of a time-series classification model, standard classification metrics such as accuracy, precision, recall, and F1-score are commonly used. However, additional metrics specific to time-series tasks can also be relevant. For instance, the dynamic time warping distance measures the similarity between two time-series by accounting for temporal distortions. The area under the receiver operating characteristic curve (AUC-ROC) is another metric that assesses the model’s ability to discriminate between different classes based on the receiver operating characteristic (ROC) curve. By focusing on effective feature extraction and selecting appropriate evaluation metrics, researchers and practitioners can enhance the accuracy and interpretability of time-series classification models. Some real-world examples of time-series classification problems include detecting anomalies in sensor data, classifying human activities based on accelerometer readings, predicting disease outbreaks based on historical data, or identifying fraudulent financial transactions based on transaction patterns. Addressing these challenges requires specialized techniques and models tailored for time-series classification, such as CNN-1D.

The remainder of this paper is organized as follows: Section 2 covers the related work that falls within the scope of our problem. Section 3 provides the context and methodology for the evaluation. In Section 4, we provide a description of the data pre-processing and experimental setup. In Section 5, we present all the essential background information required for comprehending the employed models, assessing the solutions that were generated, understanding tool workflows, and examining the descriptions of the embedded systems being tested. In Section 6, we evaluate the pre-trained model on a laptop and embedded systems. Section 7 encompasses the examination of experimental outcomes and the insights gained from the process. Finally, we conclude this paper in Section 8.

## 2. Related Work

In recent years, ESR has garnered significant attention, with a focus on leveraging DL methods to enhance the performance of ML models. Previous studies have explored various techniques for sound identification using DL models. Some models employ spectrograms or MFCCs as inputs for ANN (Artificial Neural Network) or CNN (Convolutional Neural Network) classifiers, while others directly use the raw waveform [9,10].

The authors of [11] proposed the use of a CNN for sound classification in a Wireless Sensor Network (WSN) consisting of RPi nodes. The RPi devices were utilized to encode audio and transmit it to a computer for classification. The authors demonstrated that sound classification and feature extraction could be performed on the embedded device, which possessed sufficient computational power for these tasks. Experiments were conducted by running all the necessary sound recognition operations on an RPi.

In [6], several traditional ML algorithms were implemented and evaluated on an RPi in terms of accuracy and classification time. The study introduced a hierarchical approach to audio classification, involving multiple stages and providing a flexible solution. This approach allowed for the selection of suitable classifiers for each stage, considering factors such as audio type, execution time, and power usage. In our research, we have almost a similar representation of the process, but we have made several changes and additions to incorporate operations and methods that result in improved accuracy and faster classification time compared to the previous methods.

The research presented in [5] was one of the early investigations into the application of CNNs for ESR and has become a highly influential reference for evaluating the performance of more recent networks, particularly when using the ESC dataset [12]. However, there is a lack of discussion regarding the advantages and practicality of integrating machine learning techniques for sound classification.

Within the IoT context, ref. [7] proposed a solution for the instant classification of urban sounds using a CNN model specifically designed for deployment on an RPi 4. The model utilized 2D convolutions and spectrogram feature maps as inputs for classification. However, no evaluation of the model’s inference time was provided. Similarly, ref. [8] introduced and evaluated an embedded solution that employed CNNs and spectrogram inputs for sound classification on a low-power microcontroller. Various CNN architectures were evaluated to identify the best trade-off between accuracy, CPU, and memory usage on the microcontroller. Furthermore, the timing summary, which demonstrates that lightweight Convolutional Neural Networks (CNNs) can achieve state-of-the-art accuracy even on resource-constrained embedded devices, is not provided. Meanwhile, the timing summary is integrated in our result.

In their research [13], the authors proposed a temporal–frequency attention-based CNN model (TFCNN) to effectively learn time and frequency features from Log-Mel spectrograms. To validate the effectiveness of their proposed method, they designed an experiment to examine the influence of a specific frequency band in the spectrogram on the model’s classification. Furthermore, the study introduced two novel attention mechanisms: the temporal attention mechanism and the frequency attention mechanism. These mechanisms enable the model to focus on important frequency bands and meaningful time frames within the spectrogram, effectively reducing the impact of background noise and irrelevant frequency bands.

Hardware accelerators, such as TPUs (Tensor Processing Units) and FPGAs (Field Programmable Gate Arrays), were investigated in [14] to improve the DL inference performance for audio applications. However, due to factors such as cost or real-time processing requirements, these specialized solutions may not be universally accessible or suitable for all platforms. As an alternative, CPUs were considered for inference, which can be used in both embedded devices and desktop audio plugins. The authors conducted an evaluation of the classification time and accuracy of these models, but unfortunately, they did not achieve satisfactory results on different devices. In contrast, our proposed innovation revolves around utilizing pre-trained models, which have proven to yield favorable results and can be implemented quickly.

The study [15] investigates an improved approach to multiclass damage localization, considering various damage severities and scenarios. The limited dataset challenge is addressed by augmenting the data through windowing the acceleration measurements. Additionally, a novel majority voting technique is employed using a global CNN-1D model to enhance the classification results. The effectiveness of the proposed CNN-1D model is assessed by systematically tuning hyperparameters, including window size, random weight initialization, and optimal learning rates. This evaluation showcases the robustness of the selected optimal architecture for the CNN-1D network. In our research, we faced the similar challenge of the limited dataset, and we found that augmenting the data through windowing the acceleration measurements was a good solution.

In summary, while various sound classifiers have been examined in previous research, only a limited number have been tested on embedded systems. Our evaluation goes beyond prior work by not only covering a broader range of sound classifiers but also evaluating their performance on a modern embedded system.

## 3. Methodology

This section provides an overview of sound classification and connects it with the methodology employed in this study. It then introduces the selected datasets, the pre-trained model chosen, and the metrics used for evaluation.

These experiments seek to evaluate how the performance of the pre-trained model compares across different platforms and to investigate its potential to exceed the outcomes achieved by traditional ML techniques. Similar to the many other tasks that involve classifying patterns, sound classification consists of three crucial components:

- Sensing: Quantifying a signal or audible event;
- Pre-processing of audio: Extracting distinct features from the captured sound signal;
- Lastly, classification: Identifying the sound event’s characteristics.

The audio signal processing component primarily involves extracting features from a recorded audio signal. To accomplish this, diverse methods of time-frequency analysis, originally designed for speech processing, are employed. The goal of feature extraction is to quantize the audio signal and transform it into distinct characteristic features, resulting in an N-dimensional feature vector that often represents each audio frame. A classifier then uses this feature vector to determine the context of the audio event and classify it accordingly.

The classification process involves two distinct phases:

- The training phase: During this phase, a representative model is trained through the use of pre-recorded audio data obtained from labeled training sets. In addition, during the final phase of training, a pre-trained YAMNet model was incorporated, which enabled the system to predict 521 different classes. Following this, the final dense layer was frozen and replaced with a suitable layer to meet our specific requirements. The resulting model could predict 10 classes instead of the original 512;
- The classification phase: The system obtains audio inputs from diverse sources such as pre-recorded audio data or directly from a microphone.

**Figure 1:**
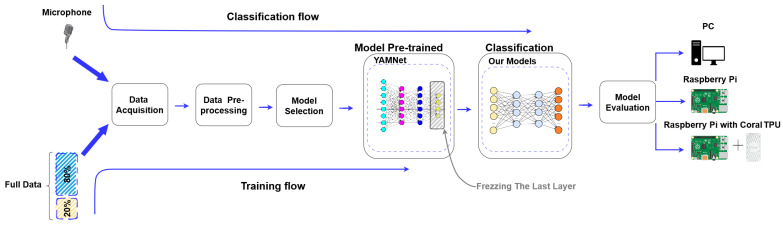
Steps involved in the process of audio classification.

During the classification stage, the models created during the training phase were employed to recognize and categorize the types of audio captured through the microphone. Figure 1 illustrates this process.

The key innovation of our method lies in the utilization of the pre-trained model in embedded systems. This approach allows us to leverage the knowledge and capabilities captured by the pre-trained model; the key innovation of our method lies in the utilization of a pre-trained model on embedded systems. This approach enables us to leverage the knowledge and capabilities captured by this model, which have been trained on large-scale datasets, and apply them to resource-constrained embedded devices. By employing a pre-trained model, we can benefit from their learned representations, feature extraction capabilities, and generalization abilities without the need for extensive on-device training or significant computational resources. This innovation enables us to achieve efficient and accurate inference on embedded systems, thereby opening up new possibilities for deploying advanced machine learning models in real-world applications with limited computational resources.

Moreover, our proposed systems distinguish themselves from previous research in the following ways:

- Implementations of the pre-trained model on embedded systems with the Coral TPU bring together several benefits, including efficient inference, real-time responsiveness, on-device privacy, portability, and edge intelligence. This integration allows for advanced AI applications at the edge, expanding the range of possibilities.
- We conducted a comparative study across different platforms, which provides valuable insights and enables us to evaluate the performance and suitability of our approach in comparison to other systems.
- During our study, we discovered that the Coral TPU is not supported on all operating systems. This finding highlights a limitation in compatibility, which should be considered when implementing our proposed systems.

### 3.1. Datasets Selection

Table 1 summarizes the characteristics of the popular datasets that we incorporated into our methodology to identify appropriate sound systems for embeddeding. Although many of these datasets encompass categories beyond urban sounds, they still encompass a significant number of representative urban sounds that align with our objective.

- BDLib2 [16]: Consisting of audio segments lasting 10 s, the collection was sourced from both the BBC Complete Sound Effects Library [17] and Freesound.org [18]. The authors carefully selected 180 samples in a meticulous manner to ensure the absence of background noise or overlap across the 10 classes. Each class comprises 18 samples, which are presented as 16-bit .wav files with a sample rate of 44,100 Hz.
- ESC [12]: In this dataset, a collection of over 2000 short audio clips are categorized into 50 different categories, which are known as ESC-50. This dataset encompasses a wide range of sounds not limited to urban environments, including animal, natural, and domestic sounds. However, for the purposes of this study, the shorter version of the dataset, ESC-10, which recognizes only 10 audio categories, was used.
- Urban Sound: This dataset is larger than other environmental sound classification datasets; it consists of over 12 GB and contains 1302 short audio clips with 10 classes with a total duration of 97,200 s of audio tagged [19]. Urban Sound was created by manually filtering and labeling each recording obtained from Freesound [19], which is an online sound repository.

### 3.2. Theoretical Background

In this section, we provide a summary of the models used in our evaluation of the two platforms. The first model is a simple ANN, and the second is a CNN, which has one-dimensional convolution layers. In our research, it was not the purpose to make two stronger models but to test pre-trained models on PC and RPi 4 with or without the USB Coral TPU.

#### 3.2.1. Artificial Neural Network

**Figure 2:**
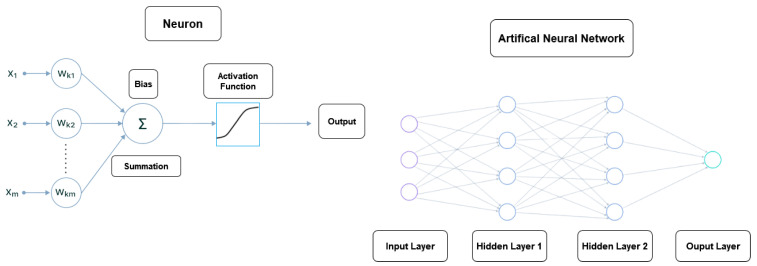
The left panel displays a node example in a neural. The right panel shows an example of an ANN with a single output. The middle nodes within the hidden layers and the last node within the output layer represent neurons.

ANN is widely recognized as one of the prevailing ML methodologies utilized in modern times [20]. The objective of this algorithm was to address intricate non-linear problems. ANNs were designed to emulate the neural networks observed in the human brain [21]. Figure 2 on the left depicts a simple example of a Neural Network architecture.

The architecture of an ANN consists of an input layer, one or more hidden layers, and an output layer [22].

As illustrated in the right panel of Figure 2, each layer consisted of one or more neurons.

In our ANN model experiment, we employed the Adam optimizer and conducted 100 epochs for each analysis. The batch size was set to 32 uniformly. The Rectified Linear Unit (ReLU) activation function was used for the first two layers, while the Softmax activation function was applied to the last layer of each model. The characteristics of our proposed ANN architecture are summarized in Table 2. The architecture consists of the following layers:

- Dense Layer 1: This layer consists of 256 neurons with the ReLU activation function.
- Dense Layer 2: This layer consists of 128 neurons with the ReLU activation function.
- Flatten Layer: This layer flattens the output from the previous layer.
- Output Layer: The final output layer consists of the output units, which are equal to the number of classes used in the dataset. The Softmax activation function is applied in the last layer.

The characteristics of our proposed ANN architecture are summarized in Table 2.

#### 3.2.2. Convolutional Neural Networks

The CNN model has made significant strides in computer vision, recognition and language modeling, among other fields [23]. Empirical evidence confirms the superior efficiency of CNN-based architectures compared to conventional methods across various classification tasks [24]. In recent years, CNNs have yielded excellent results in automatic sound event recognition. There has been a remarkable surge in the use of CNNs for classifying distinct audible sounds over the past decade [25], with numerous researchers implementing different techniques on CNNs to develop their sound classification models [26,27].

In our CNN model experiment, we incorporated several important parameters and mechanisms. These include utilizing the Adam optimizer, conducting 100 epochs for each analysis, maintaining a uniform batch size of 32, applying an L2 norm regularizer with a value of 0.001, utilizing the Rectified Linear Unit (ReLU) activation function for the initial two layers, and employing the Softmax activation function for the final layer in each model. These characteristics define our proposed architecture for the CNN model.

Hyperparameter tuning such as Adam, batch-size 32, epochs 100 and L2 norm regularizer with a value of 0.001 is a widely employed strategy to improve the performance of ML and DL models. It involves an iterative process wherein the model is retrained at each iteration to discover the optimal hyperparameter values. This technique has been widely adopted by numerous compelling research studies in the field [28].

The characteristics of our proposed ANN architecture are summarized in Table 3. The architecture is composed of the following layers:

- L1: The first layer contains 256 filters with a kernel size equal to three. The regularize L2 norm with a value of 0.001 is used. The activation function utilized is ReLU.
- L2: The second layer contains 128 filters with a kernel size equal to three. The L2 norm regularizer is also used. The padding for all the feature extraction involves in this layer is “valid” and ReLU as the activation function.
- L3: Flatten the output from the previous layer.
- L4: The last layer is the 2nd dense layer. It consists of the output units. These are equal to the number of classes used in the dataset. The Softmax activation function is used in the last layer.

#### 3.2.3. Transfer Learning

Transfer learning is an ML strategy that entails adapting a model trained on one task to a related yet distinct task by leveraging knowledge acquired from a source domain and applying it to a target domain [29]. This technique is beneficial for dealing with limited or insufficient data in the target domain. Transfer learning has been successfully employed in various domains [30], including classification, regression, and clustering. For instance, utilizing features extracted from ImageNet images has shown efficacy in the PASCAL VOC dataset [31].

In the context of sound classification, transfer learning can involve using image-based representations of sound, such as spectrograms and scalograms, as the common input to both the pre-trained model and the raw data [29]. To preprocess sound excerpts, they are commonly transformed into images, typically following suitable segmentation. CNN is frequently employed for this purpose by utilizing convolutional and pooling layers to extract the features. The classification layers connected to the initial segment are responsible for classifying the input. The most suitable approach for transfer learning depends on the characteristics of the specific problem and similarity between the original and target tasks.

### 3.3. YAMNet Model

Yet another Audio Mobilenet Network, or in short the YAMNet model, is a pre-trained deep neural network incorporating the MobileNetV1 (depthwise separable CNN) architecture. This model was trained with the AudioSet ontology [32], and it can predict audio events from 521 classes from more than 2 million YouTube clips [33]. The dataset comprises various classes of environmental sounds, such as laughter, barking, sirens, etc.

The YAMNet is an efficient model designed to fit a minimal delay in the processing of AI models on low-cost devices, where the computational resources are quite restrained. In the architecture of the MobileNet model [34], this model based on depthwise separable convolutions is replaced by the standard convolution. The original paper was provided by Google Inc. [34] to give more detail about this particular approach, where it was shown that the computational cost may be minimized up to nine times compared with standard convolution [5]. Furthermore, as shown in Table 4, the YAMNet model consists of 28 convolutional layers, 1 global average pooling layer, and 1 fully connected layer serving as its input and output layers. Depthwise separable convolutions and standard convolutions are sequentially stacked up to the pooling layer [33]. The convolutional layers in YAMNet utilize ReLU activation functions and incorporate a batch normalization technique [34]. Finally, the output layer utilizes a Softmax activation function to provide the sound class prediction [9].

**Figure 3:**
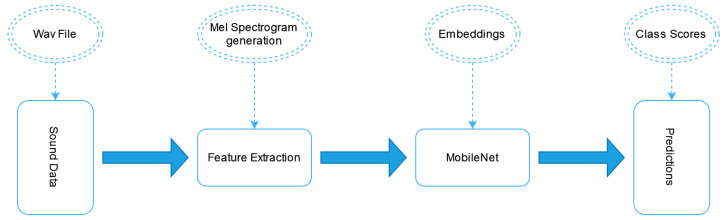
The process of conducting inferences using YAMNet was derived from a modified source.

The model accepts a 1D arbitrary length containing a waveform, and it is necessary to resample the audio clips to 16,000 HZ in the range [−1.0, +1.0] with single-channel audio [35]. The complete inference process of YAMNet, starting from the sound file and leading to the prediction, is illustrated in Figure 3.

### 3.4. Mel Spectrogram Features

The feature maps utilized as input for YAMNet are generated by extracting coefficients from time-frequency representations of sounds. Nevertheless, the frequency content of sound is not perceived linearly by humans. It is widely noted that the auditory systems of humans are most sensitive within the 2–5 kHz frequency range [36]. Our ability to differentiate tones is more accurate in lower frequency ranges compared to higher frequency ranges, where the tones may exhibit greater similarity, assuming a consistent frequency gap between the two sounds [33]. Hence, it is easy for humans to distinguish between various sounds without much effort, such as differentiating between speech and music, the sounds of a car and truck, the quality of speech of babies and adults, various speakers, noise, and other useful sounds. We want machines to be able to classify sounds in a similar way to how humans do it effortlessly [37]. In particular, YAMNet uses a Mel spectrogram as its feature map.

The *Mel* scale and Mel Spectrogram were designed to account for the logarithmic nature of human hearing. The *Mel* scale is based on the psychoacoustic perception of pitch and can be used to convert frequencies (*f*) using Equation (1). These tools were developed based on empirical evidence and consider the way that the human ear perceives sound, which is not a linear process.

(1) $$Mel\;=\;\left\{\begin{array}{cc} f & ,\;\;f\leq 1000\;\mathrm{Hz}\\ 2595\log_{10}\left(1+\frac{f}{700}\right) & ,\;f>1000\;\;\mathrm{Hz} \end{array}\right.$$

### 3.5. Metrics

To ensure a comprehensive assessment of the various classifiers, we used a range of metrics that included both accuracy and performance. This includes the $F_{1}$ score, a widely used accuracy metric, as well as metrics such as inference time, power consumption, and energy usage, which are particularly relevant for real-time applications on embedded devices. By considering both accuracy and performance, we can make more informed and complete evaluations of the classifiers.

#### 3.5.1. Accuracy

To assess the accuracy of the multi-classifiers, we used the F1 score, which combines precision and recall into a single metric. These two metrics, which are based on the number of true positives, false positives, and false negatives, are essential for evaluating the performance of the classifiers. Using the F1 score, we obtained a more comprehensive view of the classifiers’ accuracy. *Precision* (as defined by Equation (2)) measures the classifier’s ability to correctly identify the instances of each class, while *recall* (as defined by Equation (3)) represents the classifier’s ability to find all of the correct instances for each class. These metrics can be expressed as follows:

(2) $$Precision\;=\;\frac{t_{p}}{t_{p}+f_{p}}$$

(3) $$Recall\;=\;\frac{t_{p}}{t_{p}+t_{n}},$$

where $t_{p}$ represents the number of true positives, $t_{n}$ represents the number of true negatives, and $f_{p}$ represents the number of false positives. These parameters are utilized to calculate the $F_{1}$ *score* using Equation (4)

(4) $$F_{1}\;score\;=\;\frac{2\times Precision\times Recall}{Precision+Recall}.$$

#### 3.5.2. Inference Time

For real-time audio recognition, both accuracy and speed in classification are important considerations. To evaluate the classifiers, we measured the classification time, which is the amount of time required to determine the class of an audio sample. The value of this classification time may differ based on the platform being used and can be a useful factor in selecting the target platform for a given application. The algorithm used in our study is provided in Algorithm 1.

**Algorithm 1** Inference Time Calculation  1: **procedure** calculateInferenceTime(model, $X_{\mathrm{test}}$)  2:      **Variables:**  3:         input_data, start_time, prediction_inference_time, mean_time: **float**;  4:         times: list;  5:         *i*: **int**;  6:      7:      **for** $i\leq \mathrm{length}(X_{\mathrm{test}})$ **do**  8:            $input\_data=X_{\mathrm{test}}\left[i\right]$▹ Get the input data for the current sample  9:            input_data=np.newaxis(input_data,axis=0)▹ Reshape the input to match the expected shape 10:            start_time=time.perf_counter()▹ Record the start time 11:            prediction=model.predict(input_data)▹ Perform the forward pass to get the prediction 12:            inference_time=time.perf_counter()−start_time▹ Calculate the inference time 13:            times.append(inference_time×1000)▹ Append the inference time to the list
 14:      **end for** 15:      mean_time=np.mean(times)▹ Calculate the mean inference time
 16:      std_time=np.std(times)▹ Calculate the standard deviation of inference times
 17:      **return** mean_time, std_time 18: **end procedure**


## 4. Evaluation and Platform

**Figure 4:**
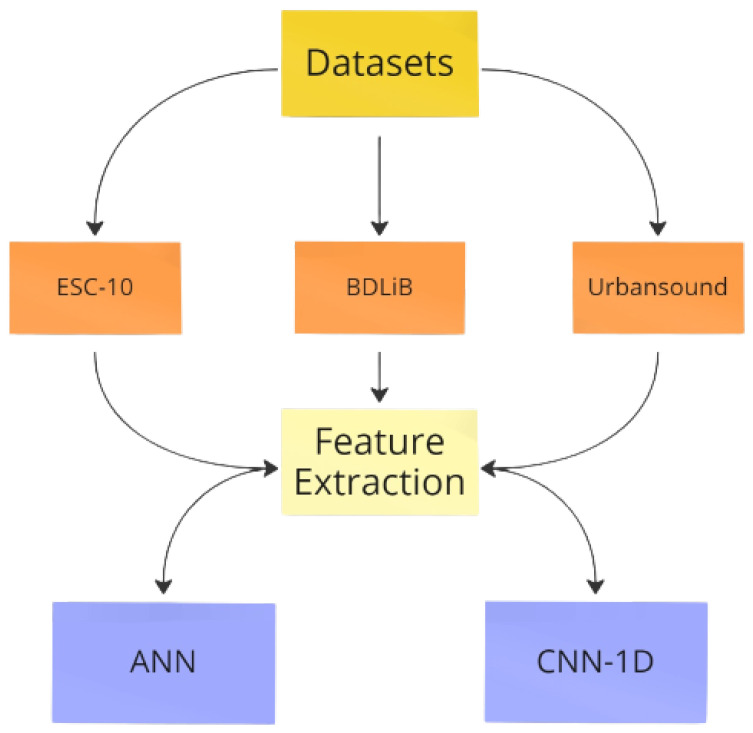
Evaluation process outlining the steps executed to ensure an equitable comparison of the DL-based approach.

In this section, the pre-processing of audio and the training procedure are provided as well as the evaluation of embedded platforms. The evaluation process as shown in Figure 4 was utilized in this evaluation. To ensure sufficient training data, a windowing technique is employed during the feature extraction process.

During this procedure, the audio features were extracted for each individual audio frame and processed sequentially. Features with similar attributes are combined to create a unified feature vector, and statistical metrics such as the mean are employed for aggregation. However, the sum, median, or GMM (Gaussian mixture model) can also be used. The purpose of this aggregation is to reduce the data and characteristics of audio frame samples into a single feature vector.

All results were then compared to a pre-trained model. The results are further detailed and discussed in the sections below.

### 4.1. Dataset Pre-Processing: Windowing

The ESC-10 dataset has 400 audio files in the ogg format compared to 180 audio files in the wav format in the BDLib dataset. Meanwhile, there are 1302 audio recordings in the Urban Sound collection. The length of the audio files in the BDLib is 10 s, while the length of those in ESC-10 is 8 s; however, the length of the audio files in the Urban Sound dataset varies from 1 s to several minutes.

To streamline the experimentation process, the audio files in the Urban Sound dataset, which are originally available in various formats such as wav, ogg, mp3, etc., are converted into the wav format. This format is preferred due to its lossless quality and the majority of audio files in the dataset already being stored in this format.

**Figure 5:**
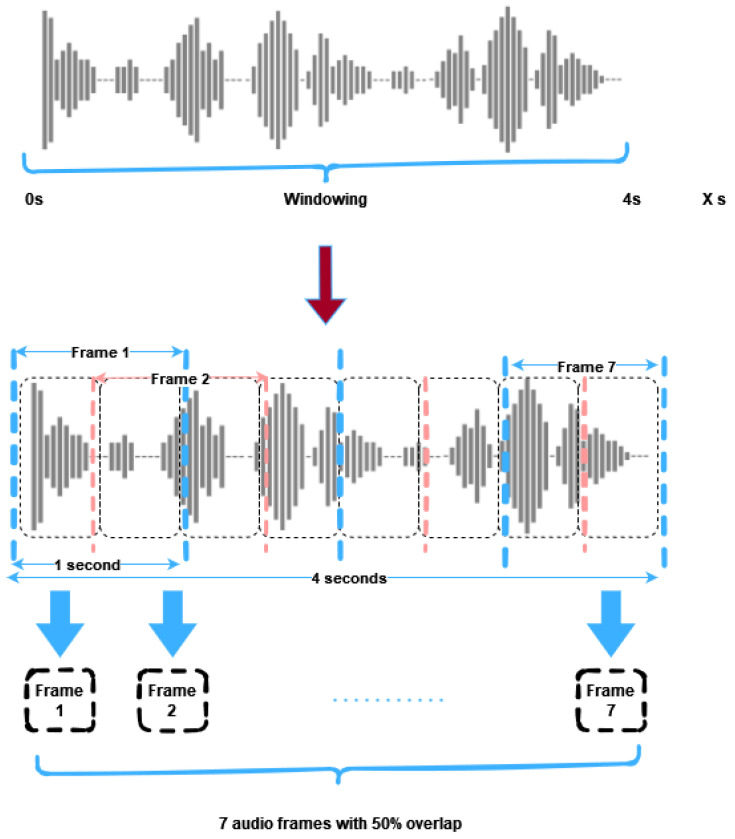
A graphical illustration depicts the utilization of the windowing method to gather seven audio segments from a four-second audio file for training purposes.

The windowing technique allows the production of additional data from these audio files. The function of the windowing process is shown in Figure 5.

The windowing flow is applied in the first 4 s when an audio file is loaded. The 4 s of audio have been split into seven separate one-second frames of audio with an overlap of 50%. The frames obtained from the windowing process are temporarily stored in an array, while features are extracted from them during this time.

The process is then repeated for each subsequent audio file in the dataset, generating seven times more feature groups to ensure an ample amount of training data. For example, in the case of the ESC-10 dataset, the windowing technique resulted in obtaining 2800 feature groups compared to only 400 without it.

It is important to note that the primary objective of windowing is to create additional training samples, and the multiple feature groups are not merged to form an averaged feature group per audio file.

The audio files in the dataset used in this research are diverse from one another, meaning that the duration of the audio files can vary. In addition, the relevant audio segments did not consistently commence at the beginning of the audio files.

Consequently, each folder of the dataset comes with the csv file content, and the metadata give all the information about the audio files, such as the start time. The starting time serves as an offset to determine the starting point of the 4 s used for windowing. For instance, if audio file X starts at 2 s, the windowing process is applied from seconds 2 to 6.

A feature group is produced for each frame, and the feature groups extracted from the initial 4 s of the audio file are utilized for both training and testing of the classifiers. This is because all the feature groups obtained from the first 4 s of the audio files are utilized to train classifiers that form the basis of our approach.

### 4.2. Dataset Processing

The classification process included partitioning the data into training (80%), testing (10%), and validation (10%) sets. Notably, feature extraction and sound classification are independent processes because they both utilize the same extracted features for classifying sounds.

Hence, the experiments in this study primarily aimed to determine the accuracy of the sound classifiers. A 5-fold cross-validation technique was employed to assess the models, where four folds were used for training with an (80%) training split and the remaining portion was divided between testing and validation.

### 4.3. Platforms and Experimental Setup

In this section, we provide an overview of the various tools and materials used in this study. The Python 3.10 package included the LibROSA 0.9.2 library, enabling effective processing and extraction of the feature. For this research, we have selected the LibROSA package to extract meaningful features from sound data. One reason for this choice is that LibROSA is compatible with Arm-based processors. We can use the librosa package in conjunction with the scikit-learn Python library, which is useful for our analysis. Additionally, the scikit-learn 1.0.2 Python library was implemented. The experiments served as a reference and were carried out on a laptop. The laptop used in the experiments featured an Intel(R) Core(TM) i7-9850H CPU running Windows 10.

One objective of this study was to execute a pre-trained model on an embedded system while maintaining high accuracy and low inference times, thus allowing for a fair comparison with laptop performance.

## 5. Optimizing Models: Toward Integration with Embedded Systems

Before initiating experimentation on embedded devices, it is critical to comprehend the limitations that ensue from the constraints imposed by the device’s design. These constraints comprise computational and power limitations that are pivotal to determining the practicality of reversing the computing process on the devices. It is pertinent to acknowledge that the majority of embedded devices operate on battery power, thereby further exacerbating the computational constraints of DL models that have become increasingly voluminous and intricate in recent years. Furthermore, the high computational demands of these models translate into substantial time requirements and power consumption.

The process of embedding models in embedded devices is crucial to adhere to a set of meticulously defined procedures. Among these procedures, one of the most pivotal involves adapting the model to the target platform, ensuring compatibility, and optimizing performance.

**Figure 6:**
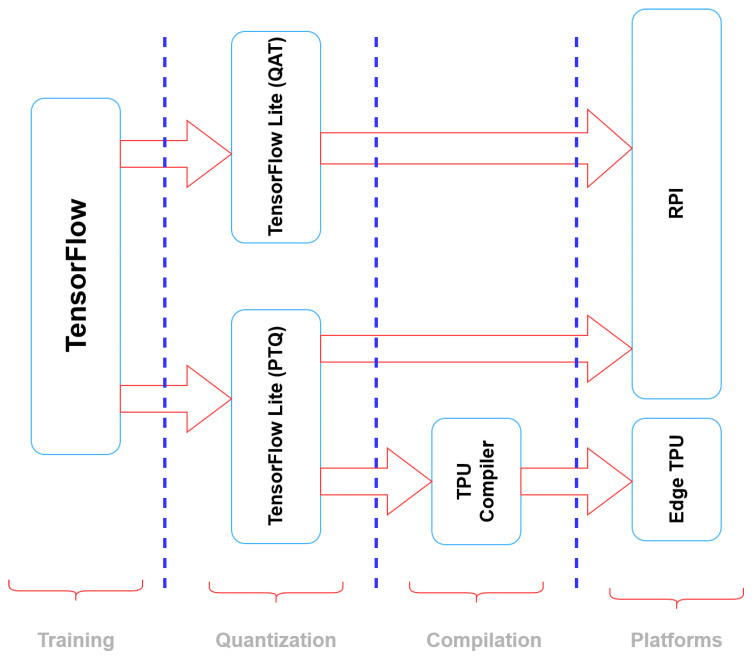
Tools for Embedding Deep Learning Models on Diverse Embedded Devices.

This procedure can be observed in Figure 6, which provides a comprehensive overview of the essential tools and techniques utilized in the process of embedding DL models onto various embedded devices. At the top of the training architecture, we employed the TensorFlow framework to train our CNN-1D model. Subsequently, we performed post-training quantization (PTQ) or quantized-aware training (QAT) to convert the floating-point models into fixed-point models. Further details regarding the specific tools used to compile the quantized models for different platforms can be found in the subsequent section. At the conclusion of our study, a comprehensive listing of the diverse embedded platforms that were employed is presented.

The TensorFlow framework, which serves as a prominent general-purpose platform, was utilized at the forefront of our results to enable the training of the CNN-1D model. Moreover, to facilitate the embedding of the model on embedded devices and mobile platforms, we also employed TensorFlow Lite (TFLite) [38], which is a specialized extension optimized for such settings. Additionally, these tools facilitate the optimization of the complexity of the model while retaining its original level of accuracy.

### 5.1. Platforms

**Figure 7:**
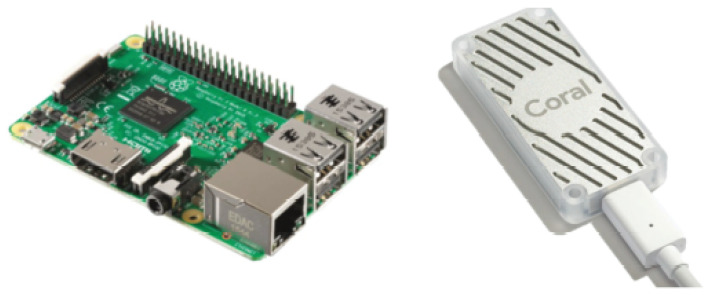
On the (**left**), we have the general-purpose embedded platform, represented by the RPi 4 B, while on the (**right**), we have the TPU-based platform, represented by the USB Coral TPU.

The general-purpose embedded platform was used to implement our models and evaluate their performance, as depicted in Figure 7.

- General-purpose embedded platform
  ✓ The RPi 4B+ is equipped with 8 GB of RAM and a quad-core 64-bit BCM 2711 SoC (ARM Cortex A72 cores) [39], which, according to previous benchmarks [40] conducted on deep learning, offers notably superior performance compared to its predecessors.
- Platform that Utilizes TPU Technology
  ✓ Google developed the USB Coral TPU, which is a dedicated hardware accelerator designed to improve the inference speed of quantized deep neural network models written in TFLite. It was initially introduced in 2017 [41] and employs a USB 3.0 interface for communication [42]. To evaluate the performance of the model, the USB Coral TPU is linked to a RPi 4B+ through a USB connection.

### 5.2. Optimizations

To achieve successful inference on an embedded platform, adjustments must be made to the DL model to accommodate differences in architecture between a laptop and the embedded platform. These adaptations serve to not only facilitate inference on the embedded platform but also optimize the performance of the model. In this subsection, the model optimization technique employed in this research will be elaborated on.

#### Model Quantization

Even though deep learning models are typically trained using floating-point numbers, fixed-point representation generally yields superior performance on embedded devices. To address this difference, the floating-point models are quantized, meaning that the model parameters are converted from floating-point to fixed-point representation. This quantization process played a vital role in optimizing the performance of our models on embedded devices.

✓ Post-Training Quantization (PTQ)
  To convert a DL model from floating point to fixed point, one common method is PTQ, which involves training the CNN-1D model using floating point and then quantizing the weights after training. It is important to note that the precision of the numbers in fixed-point representation affects the accuracy of the DL model. While it is possible for accuracy to increase, it often decreases. Additionally, the number of bits required for fixed-point representation may vary depending on the specific tool used, enabling the conversion of a 32-bit floating point DL model into a fixed-point representation with as few as 5-bit for the parameters.
  In addition to Keras, TFLite [38] provides a range of resources for converting and deploying models on embedded devices. It is primarily used for converting trained Keras models from the ‘.h5’ format to the more compact ‘.tflite’ format, which is well suited for the RPi and other embedded systems. Furthermore, TFLite offers a set of tools that can accelerate embedded inference or ensure high accuracy when using quantized models. This paper utilized TFLite v2.5.0 to perform the aforementioned tasks.

✓ Quantization Aware Training (QAT)
  QAT is an alternative approach to PTQ, where quantized parameters are utilized during the model training process. Although QAT is more time-consuming than PTQ, it is more accurate or has a smaller accuracy loss after quantization. However, it should be noted that the model error used to update the parameters during training is still calculated as a floating-point number in QAT.
  Although TF supports QAT, the model parameters are stored as 32-bit floating point numbers. Therefore, additional quantization using TFLite was necessary after training the model. Additionally, TensorFlow allows only fixed-point numbers of 8, 16, or 32-bit. Despite its greater flexibility, TFLite is the preferred option.

### 5.3. Tool Flows

The embedding of CNN models may necessitate the use of various tools and additional steps, which are platform dependent.

#### Tool Flows for TPU

**Figure 8:**
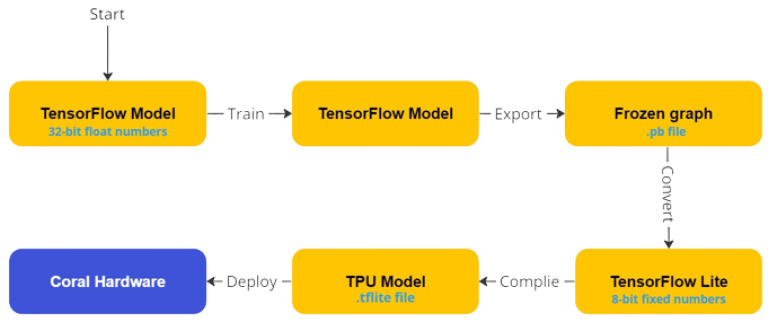
The process of embedding a model onto the Coral Dev Board can be divided into two fundamental stages. At the forefront lies the conventional training methodology, while the subsequent steps involve exporting the trained model to the boar board (emerging) at the base.

The fixed structure of TPUs imposes limitations on model optimization. To perform inference on TPU, the models must be quantized to an 8-bit integer format. The quantized models then need to be compiled for the TPU using Google’s Edge TPU compiler [43]. The Edge TPU compiler converts a “.tflite” model into a TPU-compatible format and handles the mapping of unsupported operations to the host CPU. Figure 8 depicts the tool flow used for deploying a model on a coral device.

It is a noteworthy consideration that the TPU compiler has a critical constraint, as it cannot support the execution of all operations, and consequently, some operations are offloaded to the CPU [38]. Furthermore, in the current iteration of the compiler, all operations subsequent to the initial non-supported operation are also mapped onto the CPU [44]. The CPU’s performance is inferior to that of the TPU, thereby rendering the model’s architecture a significant factor in determining its performance on the TPU [45].

## 6. Evaluating Pre-Trained Model for ESR on Various Platforms

In this section, we provide the experimental results achieved from using various platforms. The aim of this study was to ascertain whether combining a pre-trained model can lead to enhanced accuracy and a shortened inference time. Additionally, we aim to explore the advantages of a pre-trained model for embedded systems. The experimental results in this section are divided into three categories: evaluation on a laptop, assessment using an RPi, and evaluation on both the RPi and TPU platforms.

First, the ANN and CNN-1D classifiers were assessed using the BDlib, ESC-10, and Urban Sound datasets. In addition to this section, the results obtained are compared to the performance documented in the existing literature. In the second phase, three datasets were evaluated on a laptop, and the accuracy and inference time were compared to assess the potential of a pre-trained model in this context. It is widely acknowledged that model accuracy diminishes as the number of parameters increases. We employed two models: the first with a small number of parameters, as depicted in Section 3.2.1, and the second with a significantly larger number of parameters, as depicted in Section 3.2.2. Lastly, all proposed solutions were assessed on an embedded system. A solution for faster performance was found when we used an RPi with a Coral TPU.

### 6.1. Evaluation on PC

**Figure 9:**
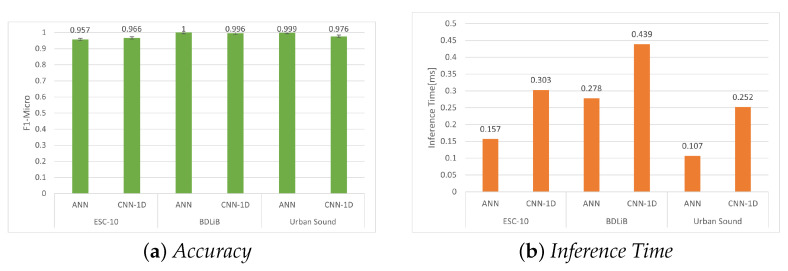
Evaluation of accuracy ($F_{1}\;micro$) and inference time for all datasets using our proposed models.

In this section, we assess the two proposed models for ESR, as described in Section 3.2. The experimental results for the ESC-10, BDLib, and Urban Sound datasets are presented in Table 5, Table 6 and Table 7, respectively. The evaluation of the BDLib dataset revealed that the ANN classifier not only outperformed the other classifiers in terms of $F_{1}$ Micro but also in other metrics. However, this superior performance comes at the expense of a higher inference time. A similar trend is observed when assessing classifiers for the Urban Sound dataset, with the ANN classifier demonstrating the highest accuracy and favorable inference time. However, the CNN-1D demonstrated higher accuracy when applied to the BDLib and Urban Sound datasets. Figure 9, shows the accuracy and inference times for all the datasets for the two models.

### 6.2. Evaluation on Embedded System

An advanced embedded system, the RPi 4B+, was employed to assess the various models. The choice of this embedded platform was driven by its ability to replicate the experiments conducted on a laptop without requiring significant modifications. Utilizing alternative embedded platforms may present challenges due to the need for proprietary tools, specialized ML support, and unique implementations when compared to open-source alternatives or different libraries. Furthermore, several traditional ML techniques may not be supported by certain platforms. The main factors to consider when applying the mentioned approaches are accuracy and computational speed. Although the accuracy is expected to remain consistent, there is likely to be a significant increase in the overall execution time. The latter is crucial for real-time recognition and relevant when selecting the audio frame size.

#### 6.2.1. Post-Training Quantization (PTQ) without Quantization

**Figure 10:**
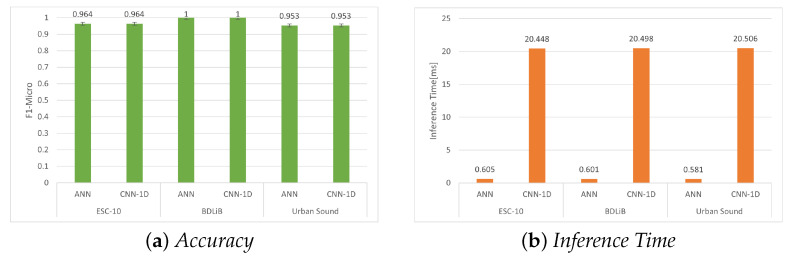
Evaluation of accuracy ($F_{1}\;micro$) and inference time for all datasets using our proposed models with post-training quantization (PTQ) without quantization.

In order to evaluate models on embedded platforms, they must be converted to TFLite [38], which is a framework specifically designed for deploying ML algorithms on embedded devices. TFLite converts a pre-trained TensorFlow model into a more compact version, enabling its deployment on embedded systems. Table 8, Table 9 and Table 10 display the performance of the full-precision TFLite models on the RPi 4B+. It is important to note that the accuracy remained consistent with the measurements obtained on a laptop. Interestingly, the inference time does not significantly change, as expected when executing on constrained embedded devices. This can be attributed to the optimization of advanced embedded devices, such as the RPi 4 B+, to efficiently support DL-based models in recent years. Consequently, there is no compromise in terms of time or accuracy when migrating models to these embedded platforms. In Figure 10, the accuracy and inference time of all the used datasets are presented.

#### 6.2.2. Post-Training Quantization (PTQ) with Quantization

**Figure 11:**
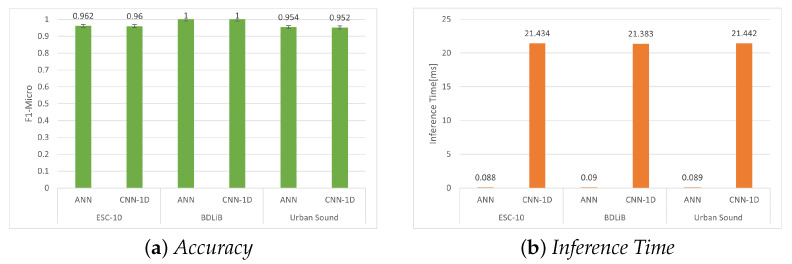
Evaluation of accuracy ($F_{1}\;micro$) and inference time for all datasets using our proposed models with post-training quantization (PTQ) with quantization.

The converted model can be either a full-precision, 32-bit floating-point model or an 8-bit integer model achieved through post-training quantization. Several constrained embedded devices or hardware accelerators, such as TensorFlow Processing Units, support only quantized models. Quantized models provide advantages in terms of reduced size and potentially improved speed compared with non-quantized alternatives. Nevertheless, the process of quantization unavoidably results in some loss of information, which can affect the accuracy achieved. Table 11, Table 12 and Table 13 present the performance of our proposed model when quantized to 8-bit integers. Despite a slight decrease in accuracy following quantization, the speed improvement is only marginally noticeable compared with the non-quantized TFLite models (Figure 11).

#### 6.2.3. Quantization Aware Training (QAT)

**Figure 12:**
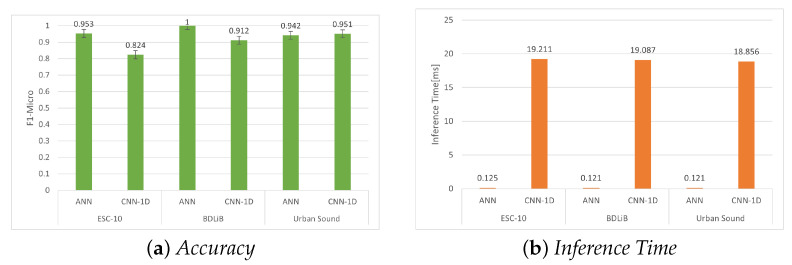
Evaluation of accuracy ($F_{1}\;micro$) and inference time for all datasets using our proposed models with quantization-aware training (QAT).

In this section, the classification accuracy of QAT 8-bit for the ANN and CNN-1D models is presented. It is evident that the accuracy of the ANN model, as shown in Table 14 and Table 15 for Esc-10 and BDLib, respectively, is slightly higher compared to the CNN-1D model. However, in Table 16, there is a slight increase in the classification accuracy for the Urban Sound dataset using the CNN-1D model (Figure 12).

### 6.3. Evaluation on RPi4 and Coral TPU

In this section, we employ quantization-aware training (QAT) to implement two models, ANN and CNN-1D, on the Coral TPU. Our objective was to evaluate the performance and accuracy of these models by utilizing Coral TPU hardware.

#### Post-Training Quantization (PTQ)

**Figure 13:**
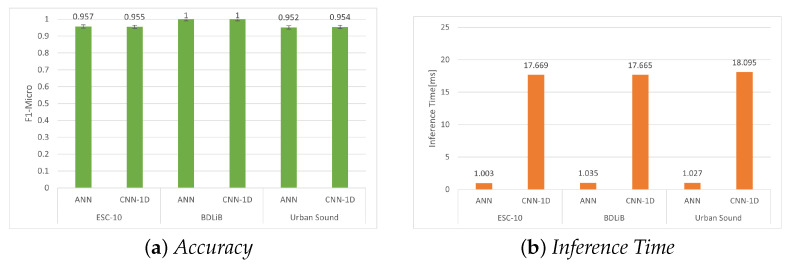
Evaluation of the accuracy ($F_{1}\;micro$) and inference time for all datasets using our proposed two models with Coral TPU and post-training quantization (PTQ).

Likewise, the two models are assessed on the embedded platform, this time with Coral TPU. While the accuracy is anticipated to be comparable to the results on the PC and RPI without Coral TPU, the execution time might improve when adapting the approach to an embedded device with Coral TPU. Table 17, Table 18 and Table 19 display the accuracy and classification times achieved when using Coral TPU in post-training quantization using 8-bit. In this case, the accuracy remained consistent with the measurements on the PC, while the classification time for the ANN model increased. However, unlike the CNN-1D standalone, where the timings decreased by three milliseconds on the RPi, the ANN model time only increased slightly, as shown in Figure 13.

### 6.4. ESR Comparison: State-of-the-Art CNNs

The effectiveness of the suggested approach was evaluated in relation to existing literature reviews that utilize the same datasets. We compared the accuracy of our proposed approach with the highest accuracy recently reported in the literature, as demonstrated in Table 20. The methodology proposed in [46] achieved the highest level of accuracy by employing Mel-Spectrogram features in conjunction with the ResNet-152 and DenseNet-161 models. In [25], two CNN models with data augmentation were presented. The authors utilized Log-Mel audio feature extraction, with the primary distinction being that the first model incorporates max-pooling while the second one does not. Unfortunately, both proposed models demonstrate increasing accuracy, but their large size imposes excessive memory demands that are impractical for embedded platforms. Furthermore, there is additional research that utilizes a similar implementation approach but employs different methodologies, unlike the one proposed by [29].

The proposed methodology significantly improves accuracy, achieving 95.70% and 96.60% accuracy, respectively, for ESC-10, 100% and 99.60% for BDLiB, and 99.90% and 97.60% for UrbanSound datasets.

Our methodology offers several anticipated advantages, including significant speed enhancements over current state-of-the-art solutions and compatibility with embedded devices due to its lower memory requirements.

## 7. Discussion

In this section below, a comparison will be made between two platforms: a 32-bit PC versus a Raspberry Pi, and a Raspberry Pi versus a Coral TPU 8-bit enabled Raspberry Pi.

The measured accuracy remained rather consistent when they are embedded. This fact can be associated with the fact that the parameters and architecture of the pre-trained model are not affected by deployment on the embedded device. According to our analysis, the accuracy of the proposed models ranged between 95 and 100% for the datasets used. This represents a higher range than the other research, as depicted in Table 20. Although Coral TPU is a new commercial device, all details are presented in Section 5.1. This facilitated the use of this technique in edge computing; however, it did not support all operations. Nonetheless, the measurement of QAT in Coral TPU is beyond the scope of this paper.

Our measurements demonstrated high accuracy in environmental sound classification. One notable implication arising from the consistent accuracy observed when deploying the models on embedded devices is the significant potential it holds for real-world applications. These findings strongly indicate that the models perform robustly and reliably, even when operating on resource-constrained platforms such as the RPi. Consequently, this opens up a wide range of possibilities for implementing sound classification systems in edge computing scenarios, where processing is conducted locally on the device rather than relying on cloud-based solutions. The ability to achieve high accuracy on embedded devices greatly enhances their suitability for diverse applications, including Internet of Things (IoT) devices, smart homes, and surveillance systems.

**Figure 14:**
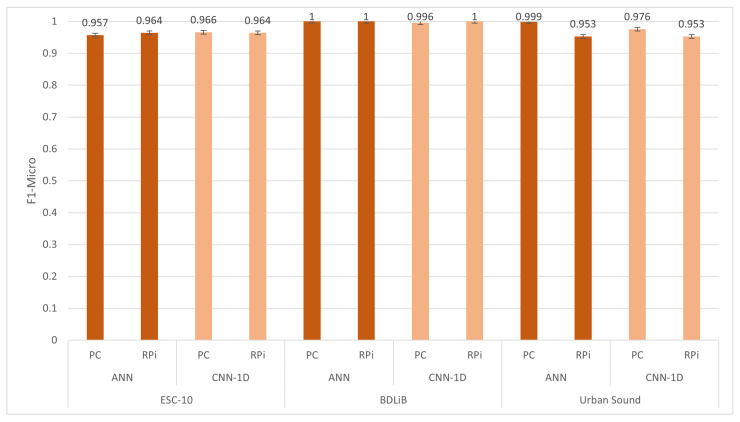
Comparison of accuracy ($F_{1}\;micro\;score$) of proposed models between PC and RPi.

**Figure 15:**
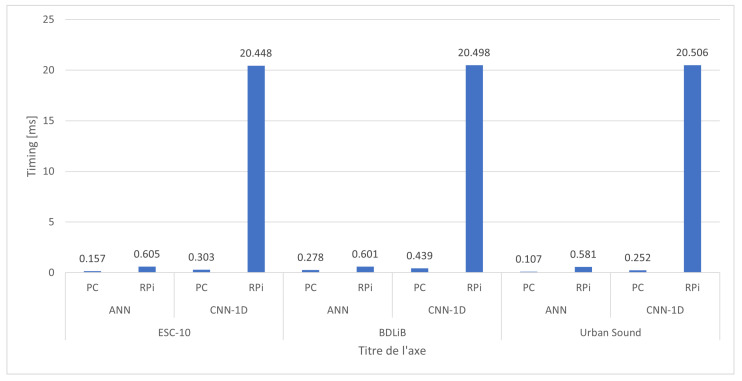
Comparison of classification time of proposed models between PC and RPi.

Additionally, we include two models proposed in our comparison in Section 3.2. The summary depicted in Figure 14 illustrates the attainable accuracies of the two distinct platforms for both models. The dependence on the dataset was significant. The ANN model achieved the highest accuracy for three datasets, while the CNN-1D model had a slightly lower accuracy compared to the ANN model. However, the performance of both models was comparable when evaluated on the BDLib dataset. The cost of achieving high accuracy with the ANN and CNN-1D models was reflected in their classification times. Figure 15 illustrates the classification and inference times for the ANN and CNN-1D models. The CNN-1D model required a significantly higher time cost to achieve this accuracy. In particular, when considering embedded platforms, the difference in performance can be as high as 20 times that of an ANN model.

The selection of audio frame size during windowing directly affects the total execution time. By considering experimental timings and assuming that audio recognition is performed in streaming mode with a 50% overlap between audio frames, the minimum windowing size is determined based on the time required for feature extraction.

The determination of shorter audio frame sizes depends on both the feature extraction time and the chosen platform. However, larger audio frame sizes can lead to power savings in embedded devices due to shorter feature extraction and classification times in comparison to the audio frame size. This observation provides invaluable insights for optimizing power consumption in resource-constrained environments. By meticulously selecting the audio frame size based on feature extraction time and platform capabilities, it becomes possible to strike a harmonious balance between accuracy and energy efficiency. This consideration assumes particular relevance in applications where power-saving modes are crucial, such as portable devices and battery-powered systems.

**Figure 16:**
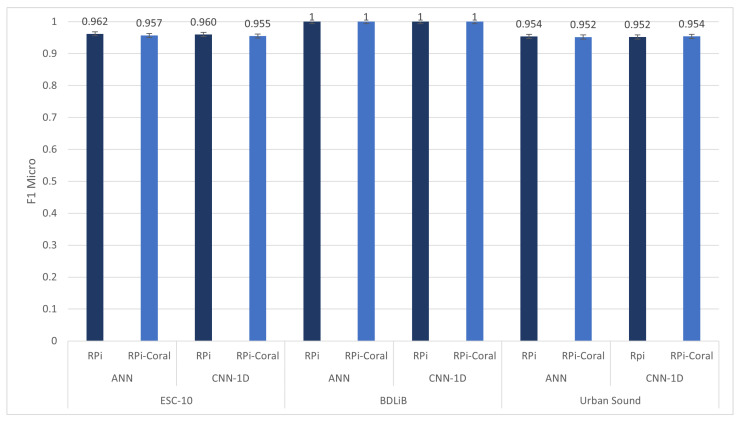
Comparison of accuracy ($F_{1}\;micro\;score$) of proposed models between RPi and RPi with Coral TPU.

Figure 16 outlines the attainable accuracies for both ANN and CNN-1D models in RPi and RPi with Coral TPU. The dependence on the dataset was quite evident. The ANN model reached the highest accuracy for three datasets, while the CNN-1D model had a negligibly lower accuracy than the ANN model. Regardless, the performances of the ANN and CNN-1D models were comparable when evaluated using the BDLib dataset. The trade-off for achieving high accuracy with these models is evident in their respective classification times.

**Figure 17:**
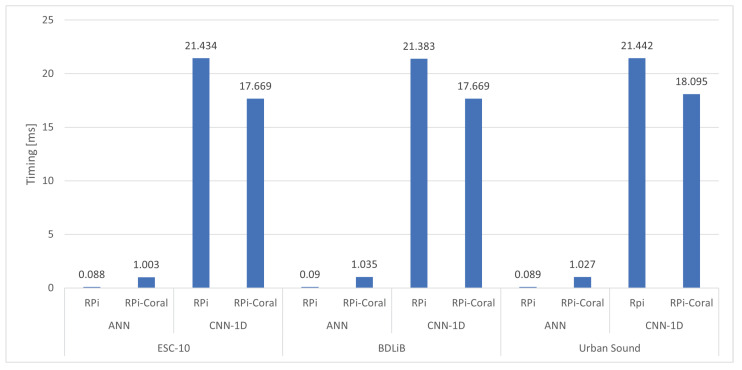
Comparison of classification time of proposed models between RPi and RPi with Coral TPU.

Figure 17 displays the classification and inference times for the ANN and CNN-1D models. The CNN-1D model requires a significantly greater time investment to achieve such accuracy, particularly when considering embedded platforms. On embedded platforms, the time cost of the CNN-1D model was up to 20 times higher than that of the ANN model.

Nevertheless, the strength of our approach lies in its ability to achieve comparable classification accuracy on embedded devices such as laptops. An important aspect that emerges from the research is the trade-off between accuracy and computational performance when comparing the ANN and CNN-1D models. Across multiple datasets, the ANN model consistently achieves higher accuracy, albeit at the cost of slightly lower accuracy for the CNN-1D model. Nevertheless, it is crucial to consider the computational cost associated with these models, especially when deployed on embedded platforms. The CNN-1D model necessitates significantly more time for classification and inference, rendering it less efficient in terms of execution time. This trade-off assumes paramount significance when selecting the most appropriate model for specific applications. If attaining high accuracy is of utmost importance and computational resources are not a constraint, the CNN-1D model may prove to be a suitable choice. Conversely, if computational efficiency is a priority, the ANN model would represent a more favorable option.

Another key finding from the research underscores the considerable impact of the chosen dataset on model performance. While the ANN model consistently achieves the highest accuracy across three datasets, the relative performance of the models varies depending on the specific dataset employed. This necessitates recognition of the fact that the models’ generalizability to unseen or diverse datasets may differ significantly. Hence, when applying these models to new sound classification tasks, it becomes imperative to carefully consider the characteristics of the specific dataset and evaluate their performance accordingly. This highlights the utmost importance of dataset selection, diversity, and augmentation techniques to ensure the models’ robustness and adaptability in real-world scenarios.

In light of these research findings, it is essential to emphasize the need for future investigations into power consumption aspects encompassing feature extraction, training, inference time, and sound classification processes. Conducting a comprehensive analysis of power consumption would engender a more comprehensive understanding of the energy requirements associated with sound classification systems. This analysis, in turn, could serve as a guide for developing power-efficient models and algorithms explicitly tailored for embedded devices. Additionally, future research should explore techniques such as quantized-aware training (QAT) to optimize model performance and resource utilization on emerging hardware platforms such as the Coral TPU.

In conclusion, the research findings offer valuable insights into the implications and limitations of deploying sound classification models on different platforms. The consistent accuracy observed on embedded devices, the trade-offs between model performance, dataset dependency, audio frame size considerations, and the focus on power consumption analysis collectively contribute to a deeper understanding of the practical applicability and optimization of sound classification systems in real-world scenarios. These findings pave the way for further advancements in the field of edge computing, wherein accurate and efficient sound classification can be achieved on resource-constrained devices.

## 8. Conclusions

The primary objective of our research is to showcase the potential of a pre-trained model to compete in terms of classification accuracy for ESR on embedded systems. Additionally, these models can attain accuracy results similar to those obtained using laptops. However, it is crucial to consider the trade-off between the increased classification time associated with these models. While this time cost is manageable on non-embedded platforms, it becomes significantly amplified when considering computational limitations on devices such as the RPi.

The results obtained indicate that pre-trained models are suitable for embedded devices owing to their resource efficiency. Nevertheless, experiments conducted on inference time revealed that larger model parameters pose difficulties for embedded devices, leading to increased inference time compared to laptops because of limited resources. Although there was a slight improvement in the inference time when applying the Coral TPU, it was negligible. Furthermore, we encountered limitations with the Coral TPU when deploying our QAT model, as it does not support all operations.

## Figures and Tables

**Table 1:** The original datasets that were used in this study.

BDLib Dataset		ESC-10 Dataset		Urban Sound Dataset	
**Categories**	**Total Time (s)**	**Categories**	**Total Time (s)**	**Categories**	**Total Time (s)**
Airplane	100	Dog barking	200	Air conditioner	6577
Alarms	100	Baby crying	200	Car horn	4819
Applause	100	Clock tick	200	Children playing	13,454
Birds	100	Person sneezing	200	Dog bark	8399
Dogs	100	Helicopter	200	Drilling	4864
Motorcycles	100	Chainsaw	200	Engine idling	3604
Rain	100	Rooster	200	Gun shot	7865
Sea waves	100	Fire cracking	200	Jackhammer	4328
Rivers	100	Sea waves	200	Siren	4477
Thunderstorm	100	Rain	200	Street music	6453

**Table 2:** Architecture of our proposed ANN model and parameter information used in each layer.

Type of Layer	Output Shape	Number of Parameters
Input_1 (InputLayer)	[(None, 1024)]	0
dense (dense)	(None, 256)	262,400
dense_1 (Dense)	(None, 128)	32,896
flatten (Flatten)	(None, 128)	0
dense_2 (Dense)	(None, 10)	1290
Total:		296,586
Trainable:		296,586
Non-trainable:		0

**Table 3:** Architecture of our CNN model and parameter details for each layer utilized in our proposed method.

Type of Layer	Output Shape	Number of Parameters
Input_1 (InputLayer)	[(None, 1024)]	0
tf.reshape (TFOpLambda)	(None, 1024, 1)	0
conv1d (Conv1D)	(None, 1022, 256)	1024
conv1d_1 (Conv1D)	(None, 1020, 128)	98,432
flatten (Flatten)	(None, 130,560)	0
dense_2 (Dense)	(None, 10)	1,305,610
Total:		1,405,066
Trainable:		1,405,066
Non-trainable:		0

**Table 4:** Details of the YAMNet model.

CNN	Type	Trained in	Number of Layers	Millions of Parameters
YAMNet	Sound	Youtube	28	3.7

**Table 5:** $F_{1}$ scores and classification times were measured for the two models using the ESC-10 dataset. The solution with the highest $F_{1}$ score is indicated.

Models	Inference Time (ms)	$F_{1}$ Micro	$F_{1}$ Macro	$F_{1}$ Weighted
ANN	0.157 ± 0.002	0.957	0.957	0.957
CNN-1D	0.303 ± 0.002	0.966	0.967	0.966

**Table 6:** $F_{1}$ scores and classification times were measured for the two models using the BDLiB dataset. The solution with the highest $F_{1}$ score is indicated.

Models	Inference Time (ms)	$F_{1}$ Micro	$F_{1}$ Macro	$F_{1}$ Weighted
ANN	0.278 ± 0.003	1.000	1.000	1.000
CNN-1D	0.4397 ± 0.010	0.996	0.996	0.996

**Table 7:** $F_{1}$ scores and classification times were measured for the two models using the Urban Sound dataset. The solution with the highest $F_{1}$ score is indicated.

Models	Inference Time (ms)	$F_{1}$ Micro	$F_{1}$ Macro	$F_{1}$ Weighted
ANN	0.107 ± 0.001	0.999	0.987	0.990
CNN-1D	0.252 ± 0.003	0.976	0.973	0.976

**Table 8:** Evaluation of RPi using TFLite without quantization on the ESC-10 dataset.

Models	Inference Time (ms)	$F_{1}$ Micro	$F_{1}$ Macro	$F_{1}$ Weighted
ANN	0.605 ± 0.024	0.964	0.965	0.965
CNN-1D	20.448 ± 0.647	0.964	0.965	0.965

**Table 9:** Evaluation of RPi using TFLite without Quantization on the BDLiB Dataset.

Models	Inference Time (ms)	$F_{1}$ Micro	$F_{1}$ Macro	$F_{1}$ Weighted
ANN	0.601 ± 0.024	1.000	1.000	1.000
CNN-1D	20.498 ± 0.575	1.000	1.000	1.000

**Table 10:** Evaluation of RPi using TFLite without Quantization on the Urban Sound Dataset.

Models	Inference Time (ms)	$F_{1}$ Micro	$F_{1}$ Macro	$F_{1}$ Weighted
ANN	0.581 ± 0.032	0.953	0.962	0.953
CNN-1D	20.506 ± 0.045	0.953	0.963	0.953

**Table 11:** Evaluation of RPi using TFLite and post-training quantization with 8-bit on the ESC-10 dataset.

Models	Inference Time (ms)	$F_{1}$ Micro	$F_{1}$ Macro	$F_{1}$ Weighted
ANN	0.088 ± 0.010	0.962	0.963	0.963
CNN-1D	21.434 ± 0.491	0.960	0.961	0.961

**Table 12:** Evaluation of RPi using TFLite and post-training quantization with 8-bit on the BDLiB dataset.

Models	Inference Time (ms)	$F_{1}$ Micro	$F_{1}$ Macro	$F_{1}$ Weighted
ANN	0.090 ± 0.016	1.000	1.000	1.000
CNN-1D	21.383 ± 0.712	1.000	1.000	1.000

**Table 13:** Evaluation of RPi using TFLite and post-training quantization with 8-bit on the Urban Sound dataset.

Models	Inference Time (ms)	$F_{1}$ Micro	$F_{1}$ Macro	$F_{1}$ Weighted
ANN	0.089 ± 0.008	0.954	0.964	0.954
CNN-1D	21.442 ± 0.365	0.952	0.961	0.952

**Table 14:** Evaluation of RPi using TFLite and quantization-aware training with 8-bit on the ESC-10 dataset.

Models	Inference Time (ms)	$F_{1}$ Micro	$F_{1}$ Macro	$F_{1}$ Weighted
ANN	0.125 ± 0.010	0.953	0.954	0.954
CNN-1D	19.211 ± 0.276	0.824	0.825	0.825

**Table 15:** Evaluation of RPi using TFLite and quantization-aware training with 8-bit on the BDLib dataset.

Models	Inference Time (ms)	$F_{1}$ Micro	$F_{1}$ Macro	$F_{1}$ Weighted
ANN	0.121 ± 0.0131	1.000	1.000	1.000
CNN-1D	19.087 ± 0.712	0.912	0.913	0.913

**Table 16:** Evaluation of RPi using TFLite and quantization-aware training with 8-bit on the Urban Sound dataset.

Models	Inference Time (ms)	$F_{1}$ Micro	$F_{1}$ Macro	$F_{1}$ Weighted
ANN	0.121 ± 0.009	0.942	0.950	0.944
CNN-1D	18.856 ± 0.171	0.951	0.960	0.951

**Table 17:** Evaluation of the ESC-10 dataset using RPi with Coral TPU and post-training quantization (PTQ) with 8-bit.

Models	Inference Time (ms)	$F_{1}$ Micro	$F_{1}$ Macro	$F_{1}$ Weighted
ANN	1.003 ± 0.405	0.957	0.957	0.957
CNN-1D	17.669 ± 2.243	0.955	0.956	0.956

**Table 18:** Evaluation of the BDLiB dataset using RPi with Coral TPU and post-training quantization (PTQ) with 8-bit.

Models	Inference Time (ms)	$F_{1}$ Micro	$F_{1}$ Macro	$F_{1}$ Weighted
ANN	1.035 ± 0.576	1.000	1.000	1.000
CNN-1D	17.665 ± 1.581	1.000	1.000	1.000

**Table 19:** Evaluation of the Urban Sound dataset using RPi with Coral TPU and post-training quantization (PTQ) with 8-bit.

Models	Inference Time (ms)	$F_{1}$ Micro	$F_{1}$ Macro	$F_{1}$ Weighted
ANN	1.027 ± 0.347	0.952	0.962	0.953
CNN-1D	18.095 ± 1.120	0.954	0.963	0.954

**Table 20:** Evaluation of Recent Sound Classifiers Based on CNNs Using Multiple Datasets.

References	Year	ESC-10	BDLib	Urban Sound
[46]	2021	94.94	-	-
[25]	2020	94.90	-	-
[29]	2021	91.25	-	-
[6]	2021	90.71	97.25	83.00
[14]	2021	83.25	74.44	63.05
Proposed ANN	2023	95.73	100	99.96
Proposed CNN	2023	96.66	99.66	97.61

## Data Availability

The data are available on demand.
